# Role of Serum Biomarkers in Early Detection of Non-Alcoholic Steatohepatitis and Fibrosis in West Virginian Children

**DOI:** 10.4172/2155-9899.1000393

**Published:** 2016-02-17

**Authors:** Komal Sodhi, Lucas Bracero, Andrew Feyh, Alexandra Nichols, Krithika Srikanthan, Tariq Latif, Deborah Preston, Joseph I Shapiro, Yoram Elitsur

**Affiliations:** 1 Department of Surgery and Pharmacology, Joan C. Edwards School of Medicine, Marshall University, USA; 2 Department of Internal Medicine, Joan C. Edwards School of Medicine, Marshall University, USA; 3 Department of Pediatrics, Division of Gastroenterology, Joan C. Edwards School of Medicine, Marshall University, USA

**Keywords:** Biomarkers, Pediatric Non-alcoholic steatohepatitis (NASH), Nonalcoholic fatty liver (NAFL)

## Abstract

**Background:**

Obesity, an epidemic among West Virginia children, as well as insulin resistance (IR), is well-established contributors to nonalcoholic steatohepatitis (NASH). Progression of NASH can lead to hepatic fibrosis and cirrhosis, making early detection imperative. The standard for diagnosing NASH is histologically *via* liver biopsy, which is highly invasive and generally contraindicated in children. By studying serum biomarkers associated with NASH, we aim to identify high risk children who can benefit from a less invasive, alternative approach to the early detection of NASH.

**Methods:**

Seventy one children were prospectively recruited and divided into 3 groups: *normal weight without IR (control), obese without IR, and obese with IR*. Serum samples were drawn for each patient and biomarker levels were assessed *via* ELISA kits.

**Results:**

*Obese without IR* and *obese with IR* patients had significantly elevated levels of lipid metabolism and accumulation markers (FGF-21, NEFA, FATP5, ApoB), oxidative stress markers (dysfunctional HDL, 8-Isoprostane), inflammatory markers(dysfunctional HDL, CK-18) and apoptosis markers (CK-18) compared to control patients (p<0.02). Bilirubin (an antioxidant) was significantly decreased in the *obese without IR* and *obese with IR* patients compared to control (p<0.02).

**Conclusion:**

This study showed a correlation between obesity, IR, and biomarkers associated with NASH in pediatrics patients from West Virginia, with obese with IR patients showing the strongest correlation. These findings support the clinical application of these serum biomarkers as a less invasive method for early detection of NASH and hepatic fibrosis.

## Introduction

Obesity levels worldwide have reached epidemic proportions, with an estimated 1.5 billion adults and 200 million school-age children around the world either overweight or obese [1,2]. Paralleling the recent increase in obesity prevalence are its comorbidities, including type 2 diabetes mellitus (T2DM), insulin resistance, and nonalcoholic fatty liver disease [3–5]. NAFLD is histologically further characterized into nonalcoholic fatty liver, which is defined as lipid accumulation present in greater than 5% of hepatocytes or affecting greater than 5% of liver volume in the absence of significant alcohol consumption, with no evidence of hepatocellular injury in the form of ballooning of the hepatocytes [6–8].

NAFLD is the most common liver disorder in the world, estimated to affect 20–40% of the population in developed nations, and up to 95% of obese people and 70% of patients with T2DM [3,6,9,10]. Though NAFL is generally asymptomatic, approximately 30% of NAFL cases progress to nonalcoholic steatohepatitis, which is histologically characterized by steatosis and inflammation with hepatocyte injury (ballooning), with or without fibrosis [11,12], and approximately 20% of NASH cases progress to liver cirrhosis [12]. NAFL and NASH are now the leading causes of chronic liver disease worldwide and NASH is expected to surpass alcohol and hepatitis as the leading cause of cirrhosis [13]. Approximately 7–14% of patients referred for liver transplantation in the United States are known to have NASH, or cirrhosis due to NASH [14].

Currently the gold standard for diagnosing NASH is *via* liver biopsy. Given the extent and burden of NAFLD in the population, liver biopsy is not logistically feasible in many parts of the country where there is limited access to health care. Percutaneous liver biopsy is highly invasive and subject to complications, including mortality, with a rate of 1 in 10,000 [15]. It is also costly and subject to sampling variability, thus, biopsy is unsuitable for longitudinal monitoring and not ideal for diagnosis [16]. Ultrasound is used by some, in conjunction with labs such as aminotransferases, to monitor progression. Ultrasound has a high sensitivity (89%) and specificity (93%) for recognizing steatosis, however its utility in detecting fibrosis has mixed results [17]. Because of all of these limitations, there has been increasing interest in finding noninvasive biomarkers to diagnose and monitor disease progression.

**Figure 1:**
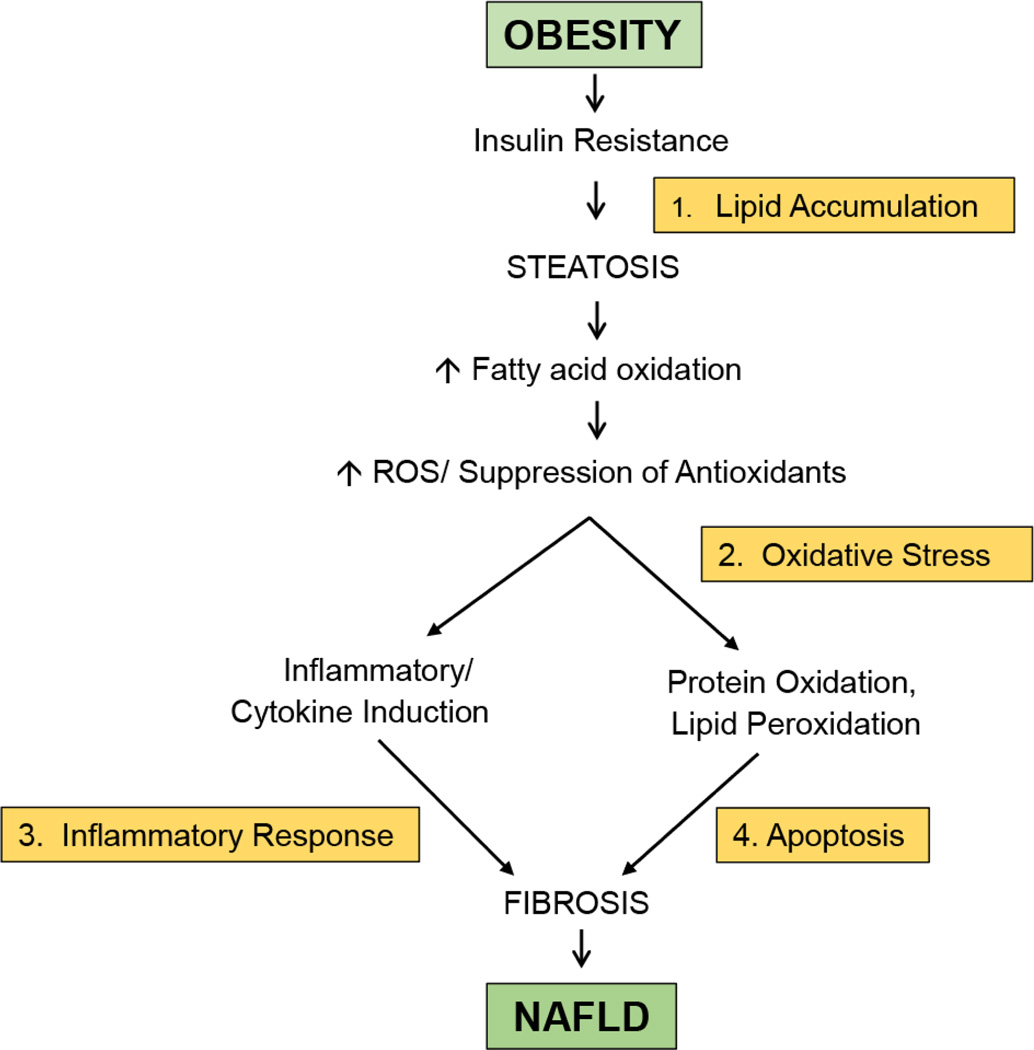
Primary mechanisms involved in the development of NAFLD. Reactive oxygen species (ROS).

Based on a review of the literature, three clinical markers and eight serum biomarkers were selected because they have shown an association with NASH in adult and/or pediatric patients. However, there is a paucity of studies within the pediatric population with regard to serological markers. This study will establish a panel of these biomarkers that will provide a minimally invasive means to detect and/or monitor NASH in pediatric patients by targeting the different mechanistic steps by which NASH develops: fat accumulation, oxidative stress, inflammation, and apoptosis (Figure 1) [15].

The goal of this panel would be to promote early detection and follow up of pediatric patients with a minimally invasive, reliable, cost effective, and logistically feasible approach in areas where obesity and its comorbidities are salient, and where health care services may be limited, such as West Virginia.

## Materials and Methods

### Patients

Children, between the ages of 8–18 years old who attended the gastroenterology clinic were prospectively recruited to the study. Exclusion criteria included children with various systemic diseases that affect the immune system such as celiac disease, inflammatory bowel disease, or children with endocrine problems including hypothyroidism, hypocalcemia, children with primary metabolic diseases (dyslipidemia, etc.), or drug induced obesity (steroids). Children were divided into 3 groups: normal weight children without IR (control), obese children without IR, and obese children with IR. The study was approved by the Joan C Edwards School of Medicine, Marshall University IRB committee.

### Blood samples and biomarker quantification

After a consent form was signed by one of the parents and the child when appropriate and after overnight fasting, venous blood was drawn from the participants and serum was stored at −80°C until analyzed. The following serum concentrations were measured by enzyme linked immunosorbent assay kits (ELISA) according to the manufacturer’s protocols: CK-18, FATP5, OxHDL/HDL and NEFA (Mybiosource, San Diego, CA); ApoB and FGF-21 (Abcam, Cambridge, MA); bilirubin (Sigma Aldrich, St. Louis, MO); 8-isoprostane(Cayman Chemical, Ann Arbor, MI). Aminotransferase (ALT) was measured by the hospital according to the International Federation of Clinical Chemistry and Laboratory Medicine standard enzymology methods. Insulin resistance was determined by the HOMA-2 equation and values >2.0 were considered positive for IR [18]. Obesity was defined by the CDC BMI growth charts for both genders (>95% tile).

### Statistics

Data were analyzed using Graphpad Prism 4.0. Equal variance was assured by Bartlett’s test for each biomarker within each of the three patient categories. ANOVA was conducted to identify statistically significant differences in the mean serum levels for the different biomarkers. The Tukey post-hoc test was used to indicate which patient groups showed statistically significant differences for the biomarker level measured.

## Results

Seventy one patients were recruited into the study and divided into three groups: normal weight without insulin resistance (n=28), obese without insulin resistance (n=16), and obese with insulin resistance (n=27). All participants were Caucasians, and male/female ratio was 1.3:1, 1.27:1, and 2:1, for the control, obese without IR, and obese with IR, respectively. There was no difference between the groups in the mean age or gender ratio. Overall, children with obesity and IR showed significant differences in almost all indices compared to normal weight children and obese children without IR (Tables 1 and 2).

### Clinical markers: BMI, HOMA-IR, ALT

BMI was significantly elevated in obese without IR and obese with IR patients, while HOMA-IR was only elevated in patients who were obese with IR, compared to the control (p<0.02) (Table 1). Alanine aminotransferase (ALT), a marker of fibrosis, was also found to be significantly higher in obese with IR patients compared to obese without IR and to control (p<0.02). These three variables are easy to obtain clinical markers that can aid in the diagnosis of NASH.

### Serum biomarkers to study lipid metabolism and accumulation: FGF-21, NEFA, FATP5, ApoB

Markers related to fat metabolism and accumulation (FGF-21, NEFA, FATP5, ApoB) were significantly elevated in the obese and obese+IR groups compared to the control group (p<0.02) (Table 2). Additionally, ApoB and NEFA were significantly elevated in the obese +IR group compared to the obese group (p<0.02). FATP5 and FGF-21 however showed no significant difference between the obese+IR and obese group.

### Serum biomarkers to study oxidative stress: Dysfunctional HDL, 8-Isoprostane, Bilirubin

Oxidative stress markers were also significantly different between the groups. Obese and obese+IR showed a significantly higher dysfunctional HDL (calculated by oxHDL to HDL ratio) compared to the control group (p<0.02). Dysfunctional HDL was also significantly higher in the obese+IR compared to the obese group (p<0.02) (Table 2). 8-Isoprostane, also a marker of oxidative stress, was significantly elevated in the obese and obese+IR groups compared to control (p<0.02). Conversely, bilirubin, which is an antioxidant compound, was significantly decreased in patients who were obese without IR and obese with IR compared to the control (p<0.02).

### Serum biomarkers to study inflammation and apoptosis: CK-18

Fragmented CK-18, a liver-specific cytoskeletal protein, is a marker for inflammation and hepatocyte apoptosis. In the obese and obese with IR patients, levels of CK-18 were significantly increased when compared to the control (p<0.02) (Table 2). Obese with IR patients showed significantly higher levels of CK-18 compared to obese without IR patients (p<0.02).

## Discussion

The goal of this study was to investigate serum biomarkers for the early detection of NASH and hepatic fibrosis in children. Currently the most reliable method of diagnosing fatty liver is histologically *via* biopsy. This method of diagnosis is invasive and because NASH lesions may be distributed irregularly throughout the liver, it can result in a false negative if unaffected tissue is biopsied or variability between lesioned samples can complicate the diagnosis [8,10,19]. Additionally biopsy is generally contraindicated in children except in rare cases where fatty liver occurs secondary to a primary liver disease [1]. For these reasons, a reliable serum biomarker panel can provide an alternative to biopsy, particularly for children in at-risk demographics for whom biopsy is not specifically indicated. In this cross-sectional study, three groups of pediatrics patients, separated according to BMI and HOMA2-IR scores, were assessed for biomarkers associated with NASH, including those related to lipid metabolism and accumulation, hepatic oxidative stress and inflammation and apoptosis.

### Lipid metabolism and accumulation

Biomarkers for lipid and carbohydrate metabolism have been associated with fatty liver. FGF-21 regulates lipid and carbohydrate metabolism by decreasing lipolysis in adipocytes, which decreases serum NEFA, and increasing glucose uptake in adipocytes *via* upregulation of GLUT-1 [20]. FGF-21 also decreases hepatic lipogenesis, upregulates enzymes for hepatic fatty acid oxidation, and has been shown to reverse hepatic steatosis in mice and non-human primates [21,22]. Although FGF-21 has beneficial effects on lipid metabolism and is protective against hepatic steatosis, fatty liver has been associated with elevated FGF-21, possibly either as compensation for increased hepatic steatosis, or because fatty liver is associated with FGF-21 resistance [22]. In our study, increased serum FGF-21 was associated with increased BMI. Previous studies have demonstrated that FGF-21 levels are increased in association with obesity and hepatic steatosis either because of unfavorable lipid and glucose metabolism associated with these conditions, or because these conditions are associated with FGF-21-resistance [22].

**Figure 2:**
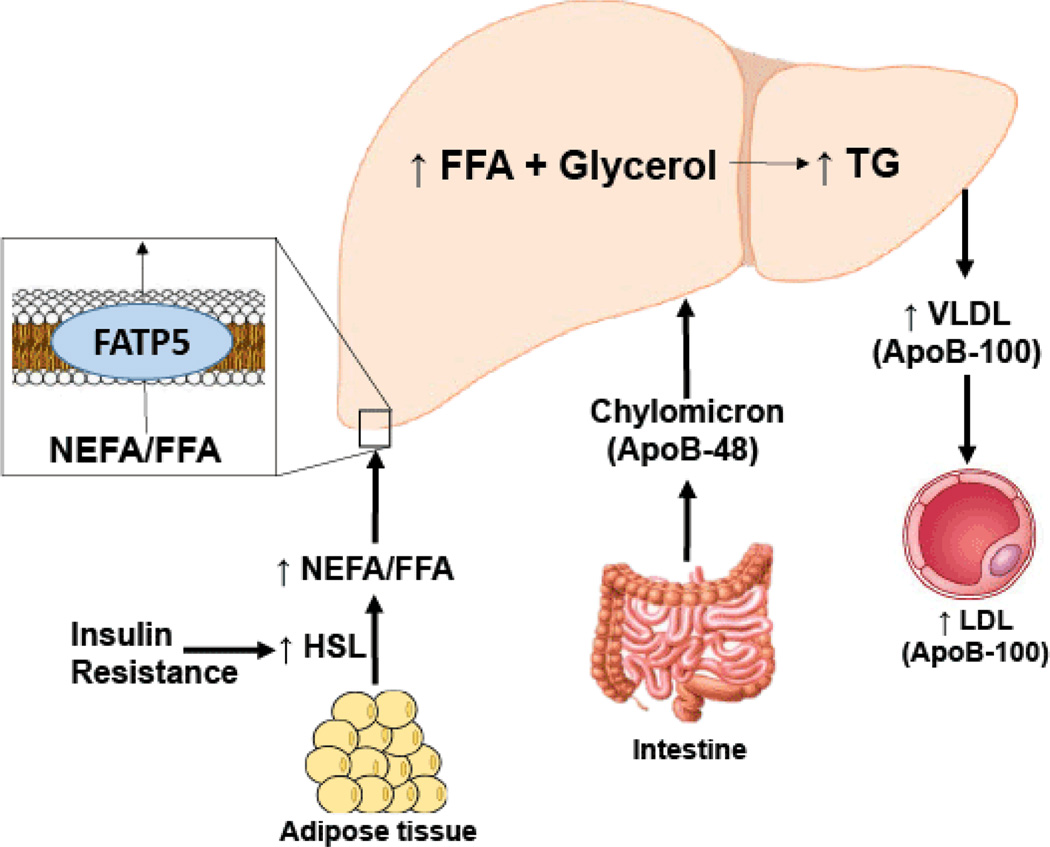
Schematic representation demonstrating lipid transport and free fatty acid (FFA) flux in insulin resistant states. Insulin resistance results in increased FFA in plasma, which leads to increased hepatocellular triglyceride (TG) concentrations. Chylomicrons, containing apoB-48, also contributes to hepatic steatosis. FFA enter the liver via fatty acid transporters (FATP), mainly FATP5. Elevated hepatic triglycerides levels leads to increased hepatocyte secretion of very low density lipoprotein (VLDL), which contains apoB-100. Insulin resistance also leads to defective hepatic mitochondrial function, resulting in decreased fatty acid oxidation in the liver.

There are several species of lipids present in the liver, but the primary form of fat is triglycerides. Based on both human and animal studies, expansion of the intrahepatic pool of FFAs is the reason for triglyceride accumulation in NAFLD (Figure 2) [23]. In patients with NAFLD, the pathways which lead to FFA efflux are usually functioning at a higher level, indicating that the critical step leading to fat accumulation is related to excessive inflow of FFA. FFA influx depends on several factors including the amount of FFA released by adipose tissue (due to insulin resistance and excessive lipolysis), dietary fat *via* chylomicron metabolism, and *de novo* synthesis (Figure 2). Studies have shown that more than half (up to 75%) of the FFA pool is derived from excess adipose tissue lipolysis [24]. Free fatty acids may be disposed of from the liver *via* fatty acid oxidation or through the assembly and export of triglycerides with VLDL.

Hepatic lipid flux involves inflow *via* NEFA and FFA through fatty acid transport protein 5 (FATP5) and outflow *via* apolipoprotein B (ApoB)-containing lipoproteins (LDL, IDL, and VLDL). Obesity and IR are associated with increased serum levels of NEFA [25–27], which have been associated with development and progression of fatty liver [28–30]. NEFA contributes directly to hepatic fat deposits [30,31], hepatic IR [25] and oxidative lipotoxic ER stress [31], which can drive NAFLD progression. Our results showed an association between BMI and IR and increased NEFA levels, which is consistent with the literature.

FATP5, a liver specific integral transmembrane protein, enhances the uptake of long chain and very long chain fatty acids. Increased FATP5 activity is associated with fatty liver [32], while decreased FATP5 activity has been shown to reverse hepatic steatosis [33]. Our results demonstrated that increases in FATP5 levels were associated with increases in BMI and IR.

ApoB, a biomarker of lipid outflow, is stimulated in response to intrahepatic triglyceride availability. Elevated serum ApoB is associated with fatty liver and has been investigated as a possible biomarker [30,34]. Though it seems that increased ApoB levels would result in net lipid clearance from the liver, it is believed that the elevated ApoB associated with fatty liver arises secondary to increased hepatic TG, as VLDL synthesis is stimulated by TG, but is inadequate in clearing the elevated hepatic lipids [30,32]. Our results indicated that compared to control, obese and IR patients were more likely to have clinical indications of net hepatic lipid influx, which is associated with hepatic steatosis: children with both obesity and IR had profiles associated with the greatest degree of net hepatic lipid influx.

### Oxidative stress

While fat accumulation is the common denominator of all forms of NAFL, an important distinction to make is that fatty infiltration alone does not lead to NASH and cirrhosis. Excess lipid accumulation results in toxic effects on hepatocytes: oxidative stress triggers inflammation and wound healing that eventually causes fibrosis [23]. Lipid accumulation and lipotoxicity can contribute to inflammatory changes that result in NASH, whereas antioxidant activity within the liver is believed to protect hepatocytes from oxidative stress [1,8,10,19,35]. Thus, oxidative stress plays a central role in hepatocyte injury and disease progression from simple steatosis to NASH. Several oxidation pathways may play a role in the overproduction of lipid peroxidation products in NASH patients which can provide quantifiable biomarkers. Furthermore, hepatic antioxidant activity is another way to detect NASH as it negatively correlates with NASH severity.

Bilirubin, an end product of heme metabolism, positively correlates with antioxidant activity, and its antioxidant properties exert a cytoprotective effect on hepatocytes. Previous studies have associated bilirubin with increased antioxidant activity via prevention of aberrant lipid oxidation and decreased oxidative lipotoxicity [36]. On the other hand, increasing ROS and oxidative stress leads to lipid peroxidation which in turn causes dysfunctional HDL [37]. oxHDL is a product of aberrant lipid oxidation and is a dysfunctional form of HDL that negatively correlates with antioxidant activity. Certain conditions, including high fat diets and diabetes mellitus, are associated with these modifications of HDL such that it paradoxically enhances LDL oxidation and/or vascular inflammation [38]. “Dysfunctional HDL” is calculated from the ratio of oxidized HDL to HDL [39]. Similarly, isoprostanes are also a marker of oxidative stress which can be measured in plasma, urine, and other biological fluids which is why they are appealing to use as biomarkers. They are prostaglandin-like compounds produced *via* the cyclooxygenase independent, free radial-catalyzed oxidation of arachidonic acid. 8-Isoprostane is a prostaglandin-F2-like compound belonging to the F2 isoprostane class [40]. 8-Isoprostane has been shown to be increased in NAFLD and NASH [41–43].

Based on a review of the literature, it appears that NASH is associated with decreased bilirubin, increased dysfunctional HDL, and increased 8-isoprostane levels, indicating increased hepatocyte susceptibility to oxidative stress [42,44]. Within this study, bilirubin levels decreased, dysfunctional HDL levels increased, and 8-isoprostane levels increased in association with increases in BMI and IR, demonstrating increasing oxidative stress with increasing BMI and IR. Our results indicate that obese and IR patients are more likely to exhibit markers of oxidative lipotoxicity, a mechanism that can drive the onset and progression of NASH.

### Inflammation

Chronic systemic inflammation plays a critical role in the development and progression of NAFLD from simple steatosis, however the exact mechanisms by which inflammation leads to NASH is still unclear [44]. It is believed that insulin resistance and a chronic low grade inflammation are through to lead to the development of NAFLD in genetically predisposed individuals. The chronic inflammation in NAFLD begins within the adipose tissue and worsens with insulin resistance [45]. Several inflammatory markers, which also happen to be markers of oxidative stress and apoptosis, have been proposed as markers for NASH. Given the complicated nature of NASH progression, starting with lipid metabolism and accumulation, and leading to oxidative stress, inflammation and apoptosis, many markers are involved in multiple steps of the process and cannot be delineated so neatly. Such markers of inflammation include elevated dysfunctional HDL, increased IL-6 [46], increased leptin [47], and decreased adiponectin [47] which have demonstrated associations with NASH in the literature and our prior studies.

HDL may be viewed as a vehicle for lipids or proteins that can be either anti-inflammatory or proinflammatory, depending on its cargo, but dysfunctional HDL is proinflammatory. Dysfunctional HDL’s proinflammatory status can improve with resolution of systemic inflammation or lifestyle and therapeutic interventions [39]. CK-18 is also recognized as a marker of liver inflammation and has been added to the panel as it is both a marker of inflammation and apoptosis [15,48]. In our studies, both dysfunctional HDL and CK-18 levels were significantly elevated in the obese and obese with IR patients compared to control and in the obese with IR compared to the obese patients.

### Apoptosis

Apoptosis, or programmed cell death, has emerged as an important mechanism in disease progression of NASH. Apoptosis is a highly organized process that can occur *via* the extrinsic or intrinsic pathway. Both pathways can lead to the activation of caspases, which cleave intracellular substrates, including cytokeratin-18 (CK-18), which is the major intermediate filament protein in hepatocytes [49]. CK-18 is a liver-specific cytoskeletal protein that is cleaved by caspases during hepatocyte apoptosis, releasing fragments that are detectable in serum samples [9,50,51]. Elevated serum CK-18 fragment levels have previously been shown to positively correlate with steatosis severity, lobular inflammation, and fibrosis stage, with markedly increased levels in patients with NASH compared to those with simple steatosis [11,52–54]. Previous studies have also shown serum CK-18 fragment levels correlated positively with the severity of hepatic inflammation and hepatocyte apoptosis associated with NASH [11,50–54]. CK-18 fragments have been validated as a marker of NASH in many studies and it has even been recognized as the most promising noninvasive test for diagnosing and managing NASH in recent NAFLD guidelines [15].

According to our results, serum CK-18 fragment levels were significantly increased in the obese and obese with IR patients compared to control and significantly increased in the obese with IR compared to the obese. The clinical index of suspicion for NASH should be high in the obese with IR group as they showed the greatest increase.

In conclusion, because obesity and IR are increasing in prevalence, and because they are risk factors for the development of NASH and hepatic fibrosis, early detection and therapy is becoming increasingly important. Our study identified a panel of serum biomarkers in children that is associated with obesity and IR. Given the strong relationship between obesity, IR, and NASH, this panel could function as a NASH screening tool for at-risk children. This is particularly important because liver biopsy is generally contraindicated in children and is too invasive for repeated monitoring of disease progression. Therefore, a reliable serum panel that is capable of identifying early NASH and fibrotic activity can provide a less invasive alternative and encourage early detection in at-risk children.

### Implications

Our study investigated the clinical efficacy of serum biomarkers in early detection of NASH and hepatic fibrosis in at-risk Appalachian children. Childhood obesity, IR, and their comorbidities are reaching epidemic proportions worldwide, with rural Appalachia suffering some of the highest rates. The poverty level within the region also underscores the importance of early detection and treatment while these conditions can be managed with minimal and relatively inexpensive intervention. The results of this study can benefit medically underserved populations by providing a reliable and less-invasive clinical approach that can serve to encourage early detection and monitoring of NASH and hepatic fibrosis in these at-risk patients.

## Figures and Tables

**Table 1:** Clinical markers.

Group	Control	Obese	Obese + IR
# Pts	28	16	27
BMI	20.87 (± 0.5)	29.63 (± 1.3)*	31.81 (± 1.1)*
HOMA2-IR	1.14 (± 0.7)	1.29 (± 0.1)	3.44 (± 0.2)*#
ALT	16.20 (± 0.6)	20.88 (± 4.2)	46.62 (± 16.5)*

Values represent means ± SEM.

* p<0.02 vs. control,

# p<0.02 vs. obese.

Body mass index (BMI), HOMA2-IR and serum concentrations of alanine aminotransferase (ALT) in control, obese without IR, and obese with IR patients.

**Table 2:** Serum biomarkers.

Group	Mechanistic Step	Control	Obese	Obese + IR
# Pts		28	16	27
FGF-21 (ng/mL)	Fat Metabolism	0.608 (± 0.02)	0.964 (± 0.03)*	0.971 (± 0.05)*
NEFA (µmol/L)	Fat Accumulation	0.47 (± 0.07)	0.88 (± 0.03)*	1.28 (± 0.04)*#
FATP5 (ng/mL)	Fat Inflow	8.00 (± 0.32)	9.46 (± 0.32)*	9.59 (± 0.29)*
ApoB (µg/mL)	Fat Outflow	1419.79 (± 37.07)	1713.12 (± 31.94)*	1914.59 (± 48.87)* #
Bilirubin (mg/dL)	Antioxidant	0.739 (± 0.04)	0.459 (± 0.03)*	0.430 (± 0.02)*
8-Isoprostane (pg/mL)	Oxidative Stress	7.34 (± 0.37)	10.54 (± 0.39)*	11.02 (± 0.44)*
Dysfunctional HDL	Oxidative Stress Inflammation	2382.75 (± 51.48)	3059.90 (± 114.62)*	3414.76 (± 146.04)*#
CK-18 (ng/mL)	Inflammation Apoptosis	57.10 (± 1.32)	68.22 (± 1.59)*	90.48 (± 2.14)*#

Values represent means ± SEM.

* p<0.02 vs. control,

# p<0.02 vs. obese.

Concentrations of biomarkers for fat accumulation, oxidative stress, inflammation, and apoptosis assessed via ELISA in control, obese without IR, and obese with IR patients.

## Abbreviations

NASH: Nonalcoholic Steatohepatitis
NAFLD: Nonalcoholic Fatty Liver Disease
IR: Insulin Resistance
FGF-21: Fibroblast Growth Factor-21
NEFA: Non-Esterified Fatty Acid
FATP5: Fatty Acid Transporter 5
FFA: Free Fatty Acid
Apo-B: Apolipoprotein B
oxHDL: oxidized High Density Lipoprotein
CK-18: Cytokeratin-18
BMI: Body Mass Index
ALT: Alanine Aminotransferase
HOMA2-IR: Homeostasis Model Assessment-Insulin Resistance
TG: Triglycerides
ROS: Reactive Oxygen Species
IL-6: Interleukin-6
